# A metagenomic catalog for exploring the plastizymes landscape covering taxa, genes, and proteins

**DOI:** 10.1038/s41598-023-43042-9

**Published:** 2023-09-25

**Authors:** Donya Afshar Jahanshahi, Shohreh Ariaeenejad, Kaveh Kavousi

**Affiliations:** 1 Department of Bioinformatics, Kish International Campus University of Tehran, Kish, Iran; 2 Laboratory of Complex Biological Systems and Bioinformatics (CBB), Department of Bioinformatics, Institute of Biochemistry and Biophysics (IBB), University of Tehran, Tehran, Iran; 3 Department of Systems and Synthetic Biology, Agricultural Biotechnology Research Institute of Iran (ABRII), Agricultural Research Education and Extension Organization (AREEO), Karaj, Iran

**Keywords:** Biotechnology, Computational biology and bioinformatics, Microbiology, Environmental sciences

## Abstract

There are significant environmental and health concerns associated with the current inefficient plastic recycling process. This study presents the first integrated reference catalog of plastic-contaminated environments obtained using an insilico workflow that could play a significant role in discovering new plastizymes. Here, we combined 66 whole metagenomic data from plastic-contaminated environment samples from four previously collected metagenome data with our new sample. In this study, an integrated plastic-contaminated environment gene, protein, taxa, and plastic degrading enzyme catalog (PDEC) was constructed. These catalogs contain 53,300,583 non-redundant genes and proteins, 691 metagenome-assembled genomes, and 136,654 plastizymes. Based on KEGG and eggNOG annotations, 42% of recognized genes lack annotations, indicating their functions remain elusive and warrant further investigation. Additionally, the PDEC catalog highlights hydrolases, peroxidases, and cutinases as the prevailing plastizymes. Ultimately, following multiple validation procedures, our effort focused on pinpointing enzymes that exhibited the highest similarity to the introduced plastizymes in terms of both sequence and three-dimensional structural aspects. This encompassed evaluating the linear composition of constituent units as well as the complex spatial conformation of the molecule. The resulting catalog is expected to improve the resolution of future multi-omics studies, providing new insights into plastic-pollution related research.

## Introduction

Polymer products are used worldwide, and at least 350–400 million tons are produced annually. Plastics are extensively consumed in the global economy for several reasons. Environmental pollution caused by plastics has become an intense ecological obstacle worldwide^1^. Plastics affect ecosystems, and all types of plastics are of particular concern to the health of humans and other living organisms on Earth. Many microorganisms and enzymes have been characterized for plastic degradation using metagenomic approaches^2^. The metagenomes of plastic-contaminated environments contain a wide variety of genomic contents, including bacteria, fungi, viruses, and other microorganisms. These organisms have developed adaptations that enable them to thrive in environments contaminated with plastic, and their genetic materials include genes and enzymes that facilitate the breakdown and metabolism of plastics. Uncultured techniques like shotgun metagenome sequencing have transformed pilot methods to identifying and investigating these communities^3–5^. The environmental microbiome (such as soil and marine samples) is a highly variable microbial ecosystem. Numerous microbes, including bacteria and archaea, colonize plastic contaminated environmental samples (landfill, agricultural land covered with plastic mulch, wastewater treatment plants, and all places that are exposed to the plastic particles^6–10^) and may play fundamental roles in degradation of recalcitrant synthetic polymers such as caprolactam (CPL is an organic compound with approximately five million tons global demand annually and is widely used to make plastics and nylon^11,12^), polyethylene (PE)^9,13^, polyethylene terephthalate (PET)^14–17^ polyamide (PA)^18–20^ and ester-based polyurethane (PUR)^21,22^, etc^23–25^.

Enzymes represent vital biological catalysts with diverse characteristics, playing a pivotal role in various biotechnological applications^8,26–28^. Analysis of the microbiome affected by plastic contamination is crucial in identifying microorganisms and enzymes that enhance plastic breakdown. Catalogs of genes, proteins, and taxa facilitate taxonomy and function categorization. The Carbohydrate-Active Enzymes (CAZy) database is a valuable and comprehensive source of enzyme information. As plastic-degrading enzymes primarily fall under the category of carbohydrate-active enzymes (CAZymes), investigation of these clusters could provide insights into plastic-degrading enzyme abundance. Enzymes identified in the degradation of polymers have belonged to PETase, MHETase, Cutinase, Laccase, Manganese Peroxidase, Lipase, Carboxylesterase, Hydrolase, and so on^9,13,21,29–36^.

Despite our growing knowledge of plastic-contaminated environment microbiomes, the complex functions of plastic-contaminated environment microbiota are still unclear. These microbiome samples were due to the absence of reference genomes and gene catalogs.

Numerous reference gene catalogs of gut microbiomes have been reported for humans^37–39^ and animals such as mice^40^, sheep^41^, chicken^42,43^, soil^44^ and camel rumen^45^. In these catalogs, the distribution of microorganisms, genes, and metabolic pathways was analyzed and investigated, depending on the need for the study.

The Study of plastic-contaminated environments is a novel research field with limited understanding of microbial diversity. To date, no integrated gene and MAGs catalog has been developed. Although some studies have examined microbial diversity in these environments, an integrated gene and taxa catalog is essential to further our understanding of this system. This will enable both taxonomic and functional profiling of the plastic-contaminated environment.

In 2017, a study reported the soil microbial diversity and functional aspects using metagenomic and bioinformatic approaches in landfill Lysimeter soil of the Ghazipur Landfill Site, New Delhi, India^46^. This study aimed to report the functional and taxonomical profiling of the Ghazipur Landfill. In 2021, Kumar et al. collected soil, leachate, and compost samples, from 10 different locations (height and depth) at the Pirana landfill site in Gujarat, India. They specified the relationship between microorganism diversity and the gene reservoirs involved in the plastic degradation process in landfill environments^47^. Another study in 2020 determined the microbial communities associated with six samples of plasticized fabric materials exposed to a harsh tropical environment for 14 months in the Republic of Panama^48^ and^49^. Metagenomic analysis of 161 Gb sequence resulted in ~ 3 million contigs and 120 MAGs. In 2017, Chu's group revealed the effects of wastewater treatment plants on the scattering of microorganisms, genes, and antibiotic-resistance genes. In July 2015, 48 samples were collected from two wastewater treatment plants in Wisconsin, USA^50^.

This study aimed to gain a better understanding of plastic-contaminated environments by combining four previously analyzed plastic-contaminated metagenomes, primarily environmental samples from Ghazipur, Gujarat, Panama, and Wisconsin, with a new soil sample collected from agricultural lands under mulch cultivation and agricultural lands irrigated with municipal wastewater.

This reseach presents a comprehensive overview of an integrated plastic-contaminated environment reference catalog that includes taxa, genes, proteins, and plastic-degrading enzymes (plastizymes). We developed this multi-functional catalog using bioinformatics tools and workflows; such as FastQC, SOAPaligner2, MEGAHIT, MetaBAT2, and MetaGeneMark. This resource is essential for gaining insights into the structure and functions of the plastic-contaminated environment. The generated catalogs can also be used to identify the characteristics of plastic-polluted environments and to attempt to remediate them for environmental conservation.

## Methods

### Metagenomic samples collection

The collection of metagenomic data from agricultural land under mulch cultivation and agricultural with municipal wastewater was important. The selected soil samples were contaminated with plastic residue for approximately 35 years. These lands are situated in Varamin and Ghaleh-No village in Tehran province with geographical coordinates (35.1848° N latitude, 51.4214° E) and (35.465° N latitude, 51.594° E).

Ten locations were sampled at various depths from the ground (5 to 20 cm), and the samples were stored in glass containers with dry ice to maintain required temperature. The collected samples were combined into one, filtered, and stored before DNA extraction. DNA was extracted from a membrane filter (cellulose ester, Millipore, Billerica, MA, United States) using a FastDNA Spin Kit (MP Biomedicals, Solon, OH, United States). The quality and quantity of the extracted DNA were assessed using agarose gel electrophoresis and a Nanodrop spectrophotometer (Thermo Scientific, Wilmington, DE, USA), respectively. For metagenome library preparation and sequencing, Illumina TruSeq DNA library preparation kit v2 (Illumina, San Diego, CA, USA) was utilized following the manufacturer's instructions. The quantity of each library was determined using a Qubit fluorimeter (Invitrogen, Carlsbad, CA, USA). All libraries were sequenced at Novogene Inc. (Beijing, China) using the Illumina HiSeq 2500 sequencing system.

We collected metagenomic data from other plastic-contaminated soil from four publicly available projects. Two samples from India SRX2861368 (n = 1) and PRJNA657696 (n = 10), one from Panama mgm4794685.3–mgm4794690.3 (n = 6), and one from the USA SRP107015 (n = 48).

### Metagenome assembly, binning, genes prediction, and construction of gene and protein catalog

After Quality control of reads by FastQC, low-quality bases (Phred score < 20) and residual Illumina adapter contaminations were trimmed and filtered by Trimmomatic software, and reads were removed by SOAPaligner2^51^. We used MEGAHIT^52^ software to assemble high-quality short reads. Options used in MEGAHIT were –kmin-1pass, -m 60e + 10, –k-min 27 –k-max 127 –k-step 10 –min-contig-len 300, -t 40. After assembly, the resulting contigs were mapped to the primitive reads using BWA^16^ to determine contig coverage profiles, and Samtools^53^ was used to convert to BAM format. Finally, we performed gene identification of the contigs from each sample by MetaGenMark^54^.

We utilized CD-HIT^55^ to construct a non-redundant gene catalog. The CD-HIT parameters were -c 0.9 -M 0 -T 0 to cluster the genes with the indicator of overlap ≥ 90%. At this step of the workflow, individual gene catalogs were constructed. These GCs were merged, and CD-HIT was used to make them unique again. Finally, we applied KofamKOALA^56^ to assign the non-redundant gene catalog to KEGG orthology^57^. eggNOG-mapper^58^ was performed to allocate clusters of orthologous groups (COG) functional categories. Also, the standalone run_dbCAN2 was used to find the presence of CAZyme genes in the final GC. To identify protein-coding regions in metagenome sequences in all contigs and each bin, we used MetaGenMark software.

### Taxonomic profiling and construction of taxa catalog

MetaPhlAn3 was used to perform taxonomic profiling of the raw data prior to assembly and contig creation. MetaPhlAn is a computational tool that assigns taxonomy to microbial communities from metagenomic shotgun sequencing. Raw reads were utilized to ascertain the abundance and diversity of the microbiome present in the sample, with respect to both bacteria and archaea. In addition, after assembly and generation of the contigs, genome bins were reconstructed using MetaBAT2 software with options –minContigLength 2000, –minContigDepth 2. The bins were merged and reduced to replicate dRep software. We first used the CheckM program^59^ to check genome bins for completeness, contamination, and strain heterogeneity. Bins with ≥ 65% completeness and ≤ 10% contamination were retained. We applied GTDB-tk to map the taxonomy to the MAGs. Finally, we used the standalone run_dbCAN2 to determine the presence of CAZyme genes in the MAGs catalog.

### Construction of plastizymes catalog

**Figure 1:**
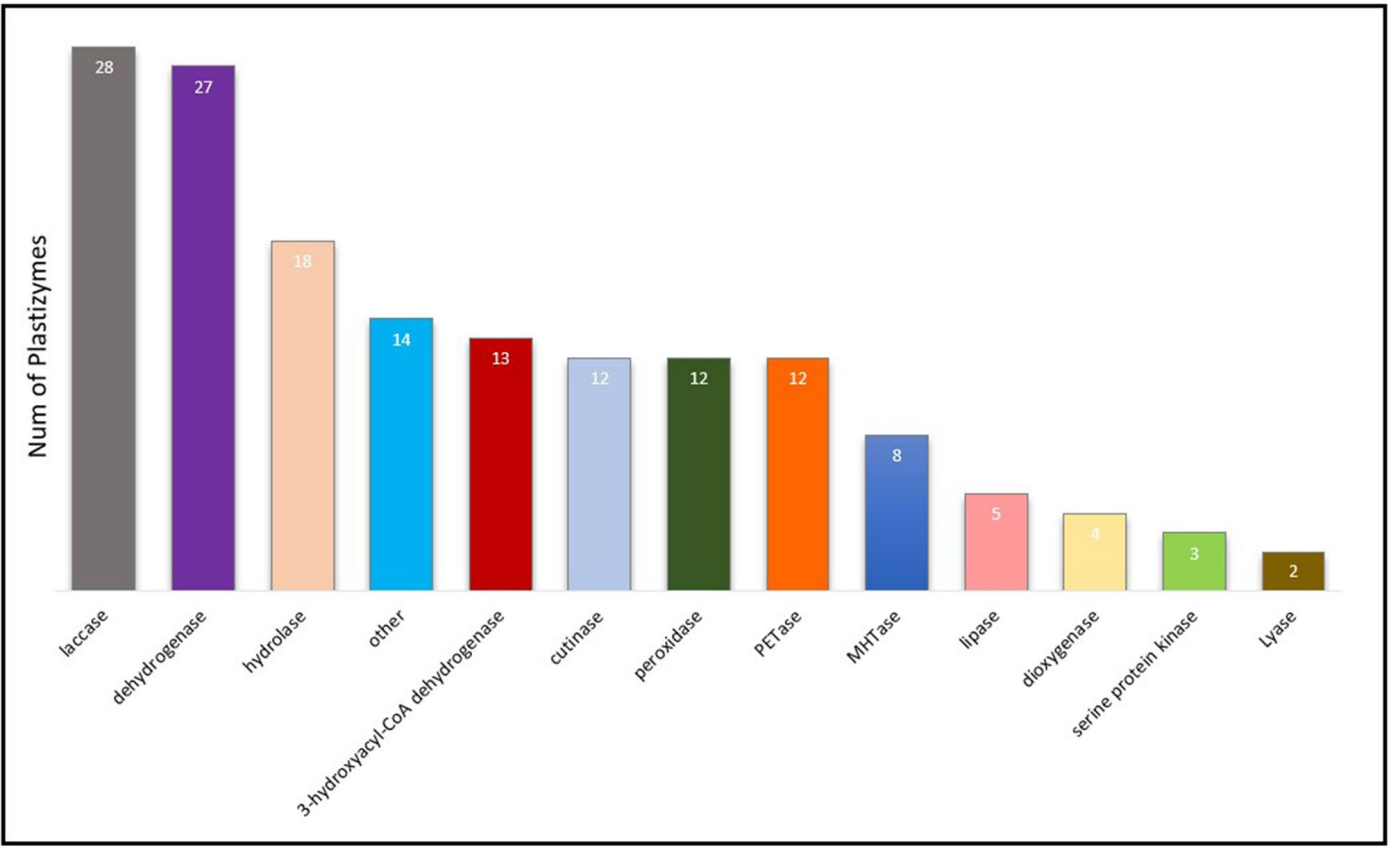
Number of plastizymes in the dataset.

We collected a new dataset consisting of 158 unique enzyme sequences with different forms of plastic degrading enzymes by tracking the NCBI’s protein database, BRENDA^60^ and UniProt^61^, reported by literature^62^. CD-HIT was utilized to reduce redundancy of highly-homologous clusters with a cut-off value of 0.9. The collected dataset contained various forms of plastizymes, including Polyethylene (PE), Polyethylene Terephthalate (PET), Caprolactam, Nylon, and others, as illustrated in Fig. 1.

Within the scope of this investigation, the MeTarEnz^63^ (metagenomic targeted enzyme miner) tool was utilized to identify putative plastic degrading genes from contigs. MeTarEnz is a multi-functional software that enables targeted screening of high-throughput metagenomic data with user-defined databases and bit-score cut-offs. A plastizyme database (158 enzyme sequences) was screened, with a minimum bit score of 200. Subsequently, the final metagenomic sequences were further analyzed using NCBI CDD^64^, Alphafold 2^65^, and TMalign^66^ tools to predict and compare the 3D structure of the predicted plastizymes.

## Results and discussion

### Construction of integrated PCEG and PCEP

**Figure 2:**
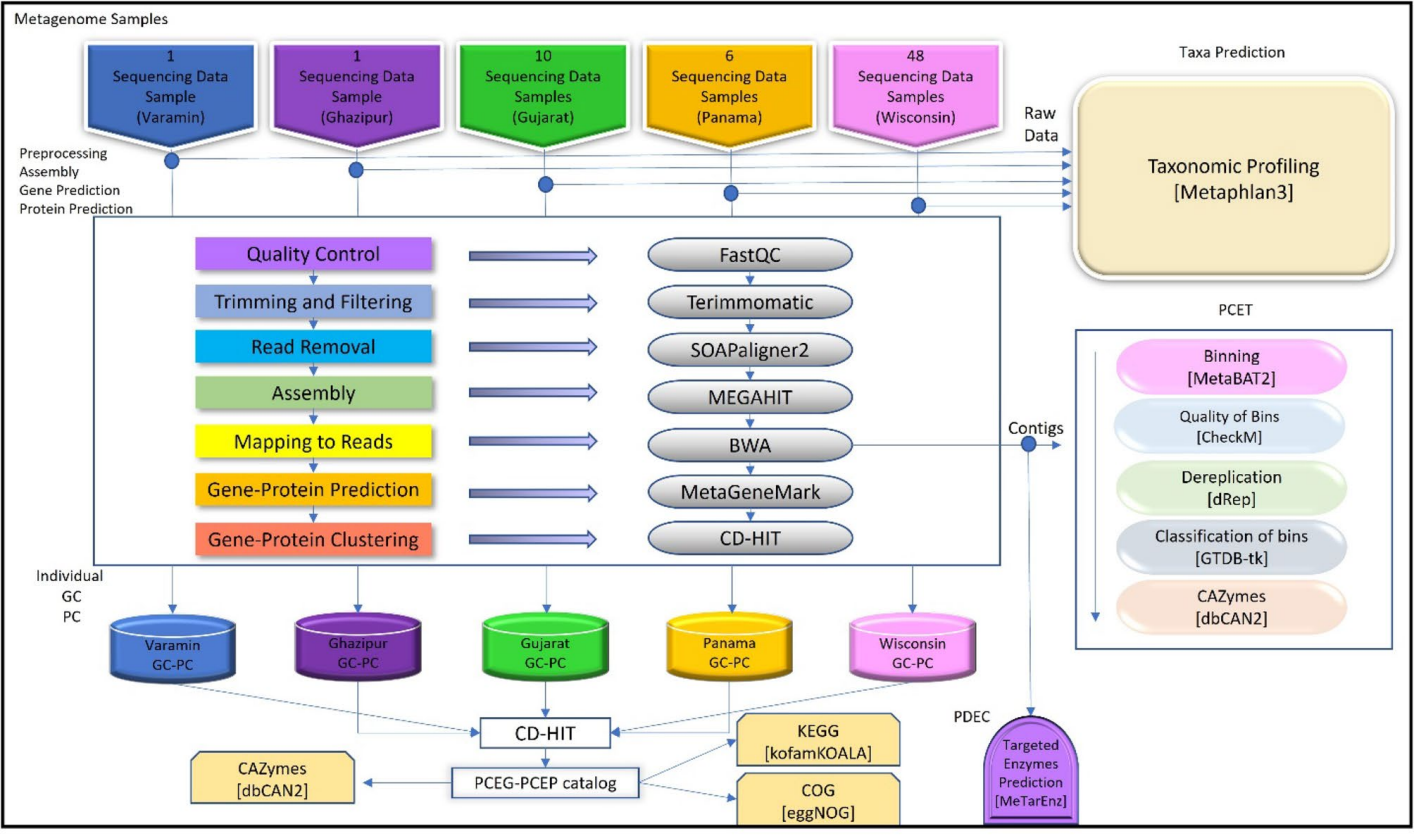
Bioinformatic workflow and tools used in data processing, analyzing, and integrating the metagenome samples. The workflow successfully processed 66 metagenome samples from five locations to generate their respective gene catalogs, which were merged into PCEG and PCEP. Taxonomic analysis with Metaphlan3 and GTDB-tk identified sequenced prokaryotic genomes or draft genomes of metagenome samples and constructed a taxa catalog. In addition, MeTarEnz was used to generate a plastizyme catalog from the contigs. The results from this workflow demonstrated the successful generation of gene and protein catalogs, taxa catalogs, and plastizyme catalogs from 66 metagenome samples from five locations.

To the best of our knowledge, the present study is the first to develop a comprehensive catalog of plastic-contaminated soils using an integrated bioinformatic workflow. Five datasets were used to produce 53,300,583 unique genes and proteins. Quality control of reads, trimming, assembly, and binning of contigs were all components of the workflow. The results of this study suggest that this workflow can be used to generate a catalog of plastic-contaminated soils and provide a basis for further investigation into the composition and function of these samples. (Fig. 2).

The proposed workflow was successfully applied to a newly plastic-contaminated soil sample combined with four previously published whole metagenome datasets. The different acquisition processes (i.e., taxonomical and functional profiling) contributed to more comprehensive results; a total of ~ 365 Gb of high-quality metagenome raw read data was obtained from the merged five datasets; this data size was greater than that of all plastic-contaminated samples. Raw reads of 66 plastic-contaminated metagenomic samples from five different locations (one sample from Varamin-Iran, one sample from Ghazipur-India, ten samples from Gujarat-India, six samples from PFHT-Panama, and 48 samples from WWTP-USA) were assembled using MEGAHIT^67^ software^68^. The assembly of short reads resulted in the identification of 44,115,301 contigs > 300 bp in length. The maximum contigs length was 954,897.

Table 1 shows the general features of each dataset. The results indicated that 53.3 million non-redundant genes and proteins were generated from the categorized complete genes. The first integrated plastic-contaminated environment gene catalog (PCEG) and integrated plastic-contaminated environment protein catalog (PCEP) were constructed. KEGG orthologs were mapped to proteins using the KofamKOALA^56^ resulting in 3,370,057 genes. Additionally, the results of the evolutionary genealogy of genes in non-supervised orthologous groups (eggnog)^58^ mapper showed that 27,718,862 genes were annotated with COG functional categories in PCEG.

**Table 1:** General feature of the individual gene catalogs.

Data set	Num of sample	Non redundant genes	Binned contigs	Unbinned contigs	Total length (bp)	Min length (bp)	Max length (bp)	Average length (bp)	References
Ghazipur	1	1,607,885	5,195	1,116,014	605,689,029	300	88,753	527	^46^
Gujarat	10	3,575,629	37,047	2,431,599	1,750,863,557	300	280,338	709	^47^
Panama	6	5,284,867	176,451	6,192,732	6,062,623,939	300	954,897	951	^48^
Wisconsin	48	41,544,363	770,174	29,972,297	16,428,178,966	300	184,708	546	^50^
Varamin	1	1,305,332	399,494	3,014,298	3,516,817,391	300	600,358	1411	In this study

**Figure 3:**
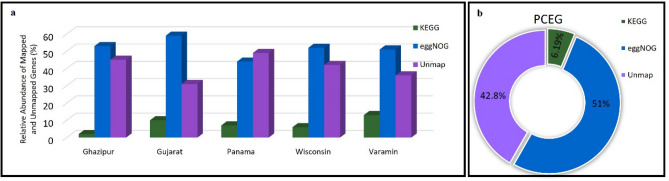
Metagenome-assembled 53,300,583 nonredundant genes from 66 plastic-contaminated soil metagenome samples. (**a**) Number of genes mapped to eggNOG^58^ and KEGG^56,57^ in the individual gene catalog and PCEG. PCEG is an integrated gene catalog produced. (**b**) Distribution of the mapped gene by kofamKOALA (KEGG), eggNOG mapper, and unmapped genes in PCEG.

These two approaches were used separately for the five individual samples and PCEG. Figure 3a displays various genes annotated by eggNOG and KEGG for all five samples, whereas Fig. 3b illustrates the percentage of genes mapped by eggNoG and KEGG in PCEG.

The results of the present study showed that the number of genes mapped to eggNOG was higher than those mapped to KEGG in all samples. This finding was in line with those of other studies, such as the construction of a gene catalog of the chicken gut microbiome^42^. The highest frequency of unmapped genes (not mapped to any mapper database) was observed in the Panama sample (48%), followed by that in the Ghazipur sample (45%). The lowest frequency was observed in the Gujarat sample (approximately 30%).

Furthermore, the Carbohydrate-Active Enzyme (CAZy) database is a powerful source of enzyme information. The findings of this research have the potential to advance the research in the field of plastic-contaminated gene catalogs^69^. To the best of our knowledge, the scattering of different groups of plastizymes in CAZyme has not been extensively investigated. Plastizymes play an important role in the hydrolysis and decomposition of plastics; therefore, understanding the abundance of these enzymes in contaminated environments is essential. Therefore, we analyzed PCEG with dbCAN2^70^ for CAZyme profiling in a sample from plastic-contaminated environment. dbCAN2 resulted in 205,066 CAZyme-encoding genes in PCEG, belonging to 51 CAZyme subclasses. The glycoside hydrolase (GH) class was the most abundant in the plastic-contaminated environment, followed by glycosyltransferase (GT) and carbohydrate-binding module (CBM). Notably, the AA, CBM, and GH groups with the AA1, AA3, and GH1 subclasses are known to be highly related to plastizymes.

**Figure 4:**
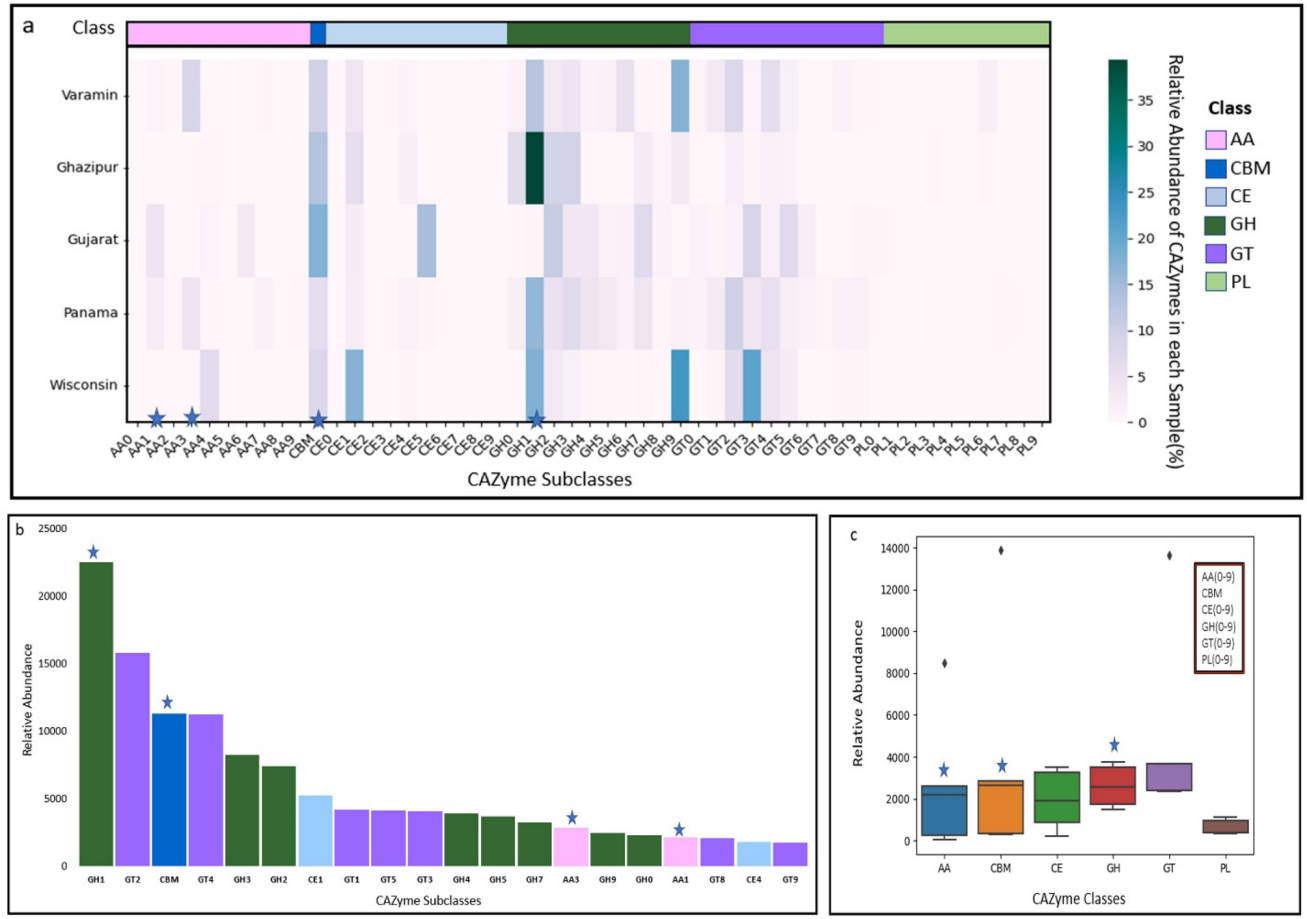
Abundance of CAZymes in plastic-contaminated soil metagenome samples. (**a**) Variations in abundance of all 51 CAZyme subclasses among the five samples. Columns in the heat map represent different CAZyme subclasses, and the color of each group is scaled from white-blue to green according to the relative abundance within the samples. (**b**) The 20 CAZyme subclasses with the most significant frequency found in PCEG. (**c**) Boxplot of relative abundance of the six CAZyme categories observed in the samples. Note: The CAZyme categories and subclasses that contained the most plastizymes are marked with a blue asterisk.

Our results indicate that CAZymes were widely distributed among the five individual samples (Fig. 4). Of the 51 subclasses identified, GH1 and CBM were the most abundant among all samples (Fig. 4a). GH1 was particularly abundant in all samples except for Gujarat. AA3 was found to be the most distributed in the Varamin sample compared with the other samples.

Overall, GH1 and GT2 were the most abundant CAZymes among the different samples, as observed at the PCEG level (Fig. 4b).

The relative abundance of the six CAZyme categories (Auxiliary Activities (AA), Carbohydrate-Binding Module (CBM), Carbohydrate Esterase (CE), Glycoside Hydrolase (GH), Glycosyl Transferase (GT), Polysaccharide Lyase (PL)) among the plastic-contaminated samples is shown in Fig. 4c. CBM was the most abundant class, followed by GH and AA. These results suggest that plastic contamination affects the abundance of CAZymes in metagenome samples.

These three categories were highly abundant among the plastizymes. (In Fig. 4, categories and subclasses that include most plastizymes are illustrated by blue asterisks). These results indicate the differential properties of the plastic-contaminated environment metagenome in terms of plastic degradation.

### Construction of integrated PCET

According to the results, microbial diversity in contaminated plastic samples is diverse, with the highest abundance of plastic degrading bacterial phylum belonging to *Actinobacteria, Firmicutes, Planctomycetes, Bacteroidetes* and *Pseudomonadota* categories. In addition, *Ideonella sakaiensis, Thermobifida fusca, Pseudomonas soli, Pseudomonas jessenii, Paenibacillus, Fusarium redolens, Fusarium spp, Penicillium*, and so on^71,72^ have been reported as the most popular plastic-degrading microorganisms. The PCET was investigated using two approaches. In the first approach, after quality control of reads, MetaPhlAn3^73^ was used for taxonomic profiling of the samples, and in the second approach, samples were analyzed by GTDB-tk^74^. According to the MetaPhlAn3 assignments, the most dominant phyla in the integrated catalog were *Proteobacteria, Actinobacteria, Firmicutes, Planctomycetes, Bacteroidetes,* and *Chloroflexi.* In total, 11 classes, 23 orders, 45 families, 42 genera, and 253 non-redundant species were predicted using the MetaPhlAn3.

**Figure 5:**
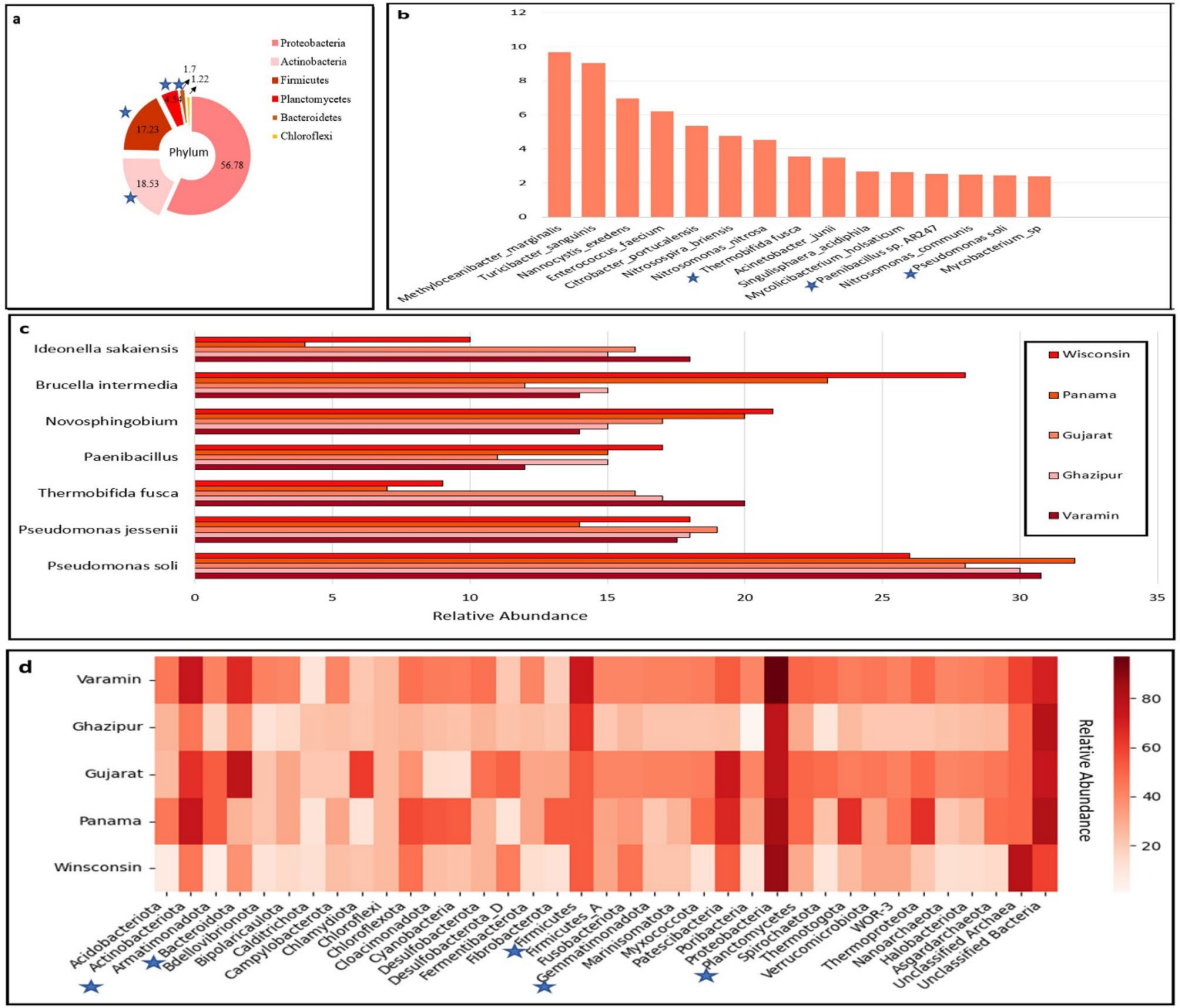
Phylum and species level taxonomic distribution. (**a**) The donut chart shows the abundance of phylum-level bacteria in PCEG (MetaPhlAn3). (**b**) The bar chart shows the distribution of the top 15 species taxonomic levels among PCET (MetaPhlAn3). (**c**) The bar charts illustrate the distribution of the top seven plastic-degrading bacterial species in all five samples (GTDB-tk and NCBI-blast). (**d**) Distribution of all 37 phylum-level taxonomic classes among PCET predicted by GTDB-tk. Note: The phylum classes and species that play the most important role in plastic degradation are marked with blue asterisks.

Figure 5a shows the relative abundance of the bacterial phyla in PCET, as determined by MetaPhlAn3. *Proteobacteria* and *Chloroflexi* were the most and least abundant phyla, respectively. *Actinobacteria, Firmicutes, Planctomycetes,* and *Bacteroidetes* were the most abundant phyla, and these groups had the highest distribution of plastic-degrading bacteria. Figure 5b shows the distribution of the top 15 species level taxonomics with the most repetition among our plastic-contaminated soil taxa catalog. Three plastic-degrading species, *Thermobifida fusca*, *Paenibacillus* sp. AR247 and *Pseudomonas* soli, were identified in the top 15 species from taxonomical profiling by MetaPhlAn3.

At this stage, several bacterial species, such as *Pseudomonas soli* and *Pseudomonas jessenii,* had the highest average integrity among all samples. (Fig. 5c). These species have been reported as the leading plastic-degrading bacteria. The results of taxonomic profiling suggested that P. soli was the most abundant plastic-degrading bacterium in all samples. Compared to other species, *Ideonella sakaiensis* had the lowest frequency. *Thermobifida fusca* and *Ideonella sakaiensis* are popular PET-degrading bacterial species. Both species had the highest frequencies in Varamin, Gujarat, and Ghazipur. The critical point is that these three environments were in direct contact with PET residues, so we can conclude that this is why these two bacterial species were more abundant compared to the other two environments. *Brucella intermedia* and *Novosphingobium*, which encode phenanthrene and caprolactam degradation enzymes, were highly abundant in the Wisconsin and Panama metagenomic samples.

In an additional analysis using the second approach, we used MetaBAT2^75^ to identify 691 individual genome bins. Among them, 97 bins were associated with a completeness score of > 65% and a contamination score of < 10%, and individual genomes, including bacteria and archaea, remained. After the dreplication of bins resulting from MetaBAT2, the number of bins generated from Ghazipur, Gujarat, Panama, Wisconsin, and Varamin was 5, 62, 122, 127, and 75, respectively. All binned samples were analyzed using GTDB-tk^74^. According to the GTDB-tk assignments, in PCET, 844 MAGs (691 non-redundant) were assigned to the 16S rRNA gene sequence. Figure 5d illustrates the distribution of all 37 phylum-level taxonomies in the prepared plastic-contaminated soil taxa catalog. *Proteobacteria* and *Calditrichota* had the highest and lowest frequencies in all five samples, respectively. *Actinobacteria*, *Bacteroidota*, *Firmicutes*, *Gemmatimonadota*, and *Planctomycetes* were the phyla most related to plastic digestion bacteria, with the highest amount found in Varamin and Panama (*Actinobacteria*), Varamin and Gujarat (*Bacteroidota*), Varamin (*Firmicutes*), Varamin and Gujarat (*Gemmatimonadota*), and Varamin, Gujarat, and Panama (*Planctomycetes*).

The most abundant bacterial class, Order, Family, Genus, and Species-level taxonomic profiling were *Bacteroidia* (21.6%), *Steroidobacterales* (20.11%), *Pseudomonadaceae* (12.22%), *Fermentibacter* (9.6%) and *Methyloceanibacter_marginalis* (9.7%). Additionally, 82 novel bacterial genomes were identified, belonging to novel species, respectively.

**Figure 6:**
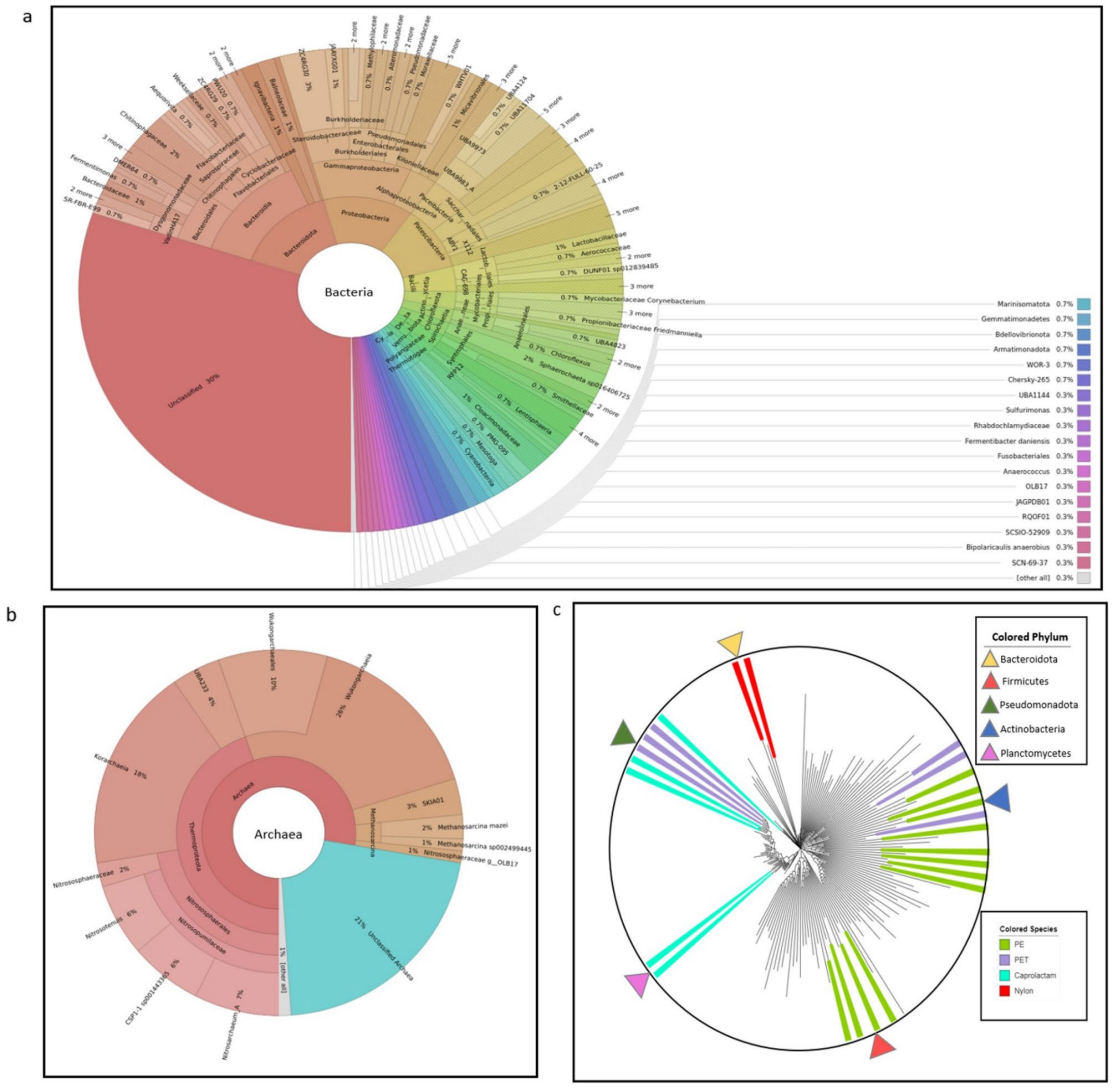
Diagrams showing the relative abundance diversity and phylogenetic tree of full-level taxonomic profiling of the 410 most abundant OTUs for the PCET. (**a**) Distribution of bacterial genomes in PCET. (**b**) Distribution of archaeal genomes in PCET. **c** Phylogenetic tree of 194 MAGs containing plastic-degrading bacteria. Colors inside the circle correspond to species with a role in caprolactam, nylon, PET, and PE-degrading bacteria. Colored triangles show the phyla of species that have the ability to digest plastic. Note: The diversity of taxa and taxonomic phylogenetic trees were visualized using the Krona chart-2.8.1^76^ (https://github.com/marbl/Krona/wiki) and Interactive Tree Of Life (iTOL) v5^77^ (https://itol.embl.de/), respectively.

GTDB-tk analysis revealed 19 archaeal genomes, of which nine belonged to the phylum *Thermoproteota* (47%). *Asgardarchaeota, Halobacteriota,* and *Nanoarchaeota* were at the phylum level. Furthermore, 18 archaeal genomes were novel species. Figure 6a, b illustrate the relative abundance of the 410 most abundant OTUs bacterial and archaeal genomes in PCET.

To date, three species of sac fungi, *chrysosporium*, *Aspergillus niger*, and *fusarium solani* have been reported to be the leading plastic-degrading microorganisms. The vital point of our results is that a significant number of these microorganisms were identified in four samples Varamin (154), Gujarat (23), Panama (44), and Wisconsin (227).

The phylogenetic tree of 194 MAGs containing plastic-degrading bacteria is shown in Fig. 6c. The main species of identified plastic-degrading bacteria belong to *Pseudomonadota* and *Actinobacteria. Brucella intermedia, Novosphingobium, Pseudomonas soli, Ideonella sakaiensis, and so on* belong to members of *Pseudomonadota*. *Streptomyces clavuligerus, Paenarthrobacter ureafaciens,* etc., belong to members of *Actinobacteria*. (Fig. 6c; Table 2).

**Table 2:** The table shows the identified species associated with plastic degradation based on detailed reports in the phylogenetic tree shown in Fig. 6 (Section c).

Name	Phylum	Type of encoded plastizymes	Type of plastic
*Brucella intermedia*	*Pseudomonadota*	Dehydrogenase	Caprolactam
*Novosphingobium sp*	*Pseudomonadota*	Phenanthrene dioxygenase	Phenanthrene
*Pseudomonas soli*	*Pseudomonadota*	3-Hydroxyacyl-CoA dehydrogenase	Caprolactam
*Ideonella sakaiensis*	*Pseudomonadota*	PETase	PET
*Acidovorax carolinensis*	*Pseudomonadota*	Laccase	caprolactam
*Azoarcus*	*Pseudomonadota*	Laccase	Phthalate-PET
*Comamonas*	*Pseudomonadota*	Laccase	Phthalate-PET
*Massilia*	*Pseudomonadota*	Laccase	Phenanthrene-PET
*Paraburkholderia caribensis*	*Pseudomonadota*	Laccase	Phenanthrene-PET
*Streptomyces clavuligerus*	*Actinobacteria*	Hydroxylase	PE
*Paenarthrobacter ureafaciens*	*Actinobacteria*	6-Aminohexanoate-dimer hydrolase	PE-nylon
*Thermobifida alba*	*Actinobacteria*	Cutinase	PET
*Thermobifida fusca*	*Actinobacteria*	Cutinase	PET
*Streptomyces sp. SM14*	*Actinobacteria*	Alpha/beta hydrolase	PET-MHETase
*Thermomonospora curvata*	*Actinobacteria*	Lipase	PET-MHETase
*Thermobifida halotolerans*	*Actinobacteria*	Cutinase	PET
*Gordonia phthalatica*	*Actinobacteria*	Laccase	PE-phthalate
*Micrococcus luteus*	*Actinobacteria*	Hydrolase	PE
*Rhodococcus erythropolis*	*Actinobacteria*	Hydrolase	PE
*Nocardia asteroides*	*Actinobacteria*	Hydrolase	PE
*Mycobacterium sp*	*Actinobacteria*	Laccase	PE-phenanthrene
*Flavobacterium*	*Bacteroidota*	6-Aminohexanoate-cyclic-dimer hydrolase	Nylon
*Cytophagales*	*Bacteroidota*	Hydrolase	Nylon
*Bacillus subtilis*	*Firmicutes*	Para-nitrobenzylesterase	PE
*Sphaericus*	*Firmicutes*	Hydrolase	PE
*Lactobacillales*	*Firmicutes*	Esterase	PE
*Brevibacillus borstelensis*	*Firmicutes*	Hydrolase	PE
*rubripirellula*	*Planctomycetes*	Peroxidase	Caprolactam
*Pirellulase*	*Planctomycetes*	Peroxidase	Caprolactam

In this study, we identified a remarkable number of bacterial species that were experimentally introduced as plastic-degrading bacteria in contaminated plastic metagenome samples collected by PCET. These findings suggest that these bacteria are capable of degrading plastic in the environment. Further research is needed to understand the role of these bacteria in plastics degradation.

### Construction of integrated PDEC

Construction of the plastizyme catalog revealed a large number of enzymes with the highest similarity to the plastizymes database in both sequence and 3D structural dimensions.

To identify possible novel plastizymes, after mapping the contigs against raw reads using BWA, the resulting contigs were screened using MeTarEnz^63^ against the plastizymes’ dataset collected in the investigation with the maximum bit score and minimum E-value. Among the analyzed data, 136,654 plastic-degrading enzyme sequences were predicted. Furthermore, to validate the results of the identified plastizymes, AlphaFold2^65^ was used to predict the 3D structure and TM-align for 3D structure comparison of some of the obtained plastizymes (approximately 700 sequences). The AlphaFold predicted local distance difference test scores (pLDDT) were greater than 80% for all predicted enzymes, and the TM scores were between 70 and 98%. These scores indicated the high affinity of the identified enzymes for plastic degradation enzymes.

Putative enzymes belong to 12 different plastizyme families, including peroxidases, PETases, cutinases, and laccases. Table 3 shows details of the main plastizymes. After applying MeTarEnz and segregating all identified plastizymes from PDEC with the highest similarity to database sequences, NCBI-CDD was used to compare the conserved domains, superfamilies, and top-hit PDB structures.

**Table 3:** List of main enzymes involved in biodegradation of all types of plastics, EC number, superfamily name, and top-hit 3D structure PDB codes identified in within the scope of this research.

Name of enzymes	EC No	Superfamily name	Top hit PDB code
3-Hydroxyacyl-CoA dehydrogenase	1.1.1.211	PRK08268	c3mogA_
Lyase	4.2.-	Lyase_I_like	d1gkma_
Serine protein kinase	2.7.11.1	PRK15455	c6iy8B_
Dehydrogenase	1.1.1.1	ALDH-SF	c4f9iA_
Hydrolase	3.1.1.-	Abhydrolase	c3visB_
Cutinase	3.1.1.74	Abhydrolase	d1cexa_
Laccase	1.10.3.2	Cu-oxidase_4	c5daoA_
Dioxygenase	1.14.-	Rieske	c2gbxE_
Peroxidase	1.11.1.-	Thioredoxin_like	c2v2gC_
Lipase	3.1.1.3	Abhydrolase	c3visB_
PETase	3.1.1.101	Abhydrolase	c5xg0A_
MHTase	3.1.1.102	Abhydrolase	c6qgbA_

**Figure 7:**
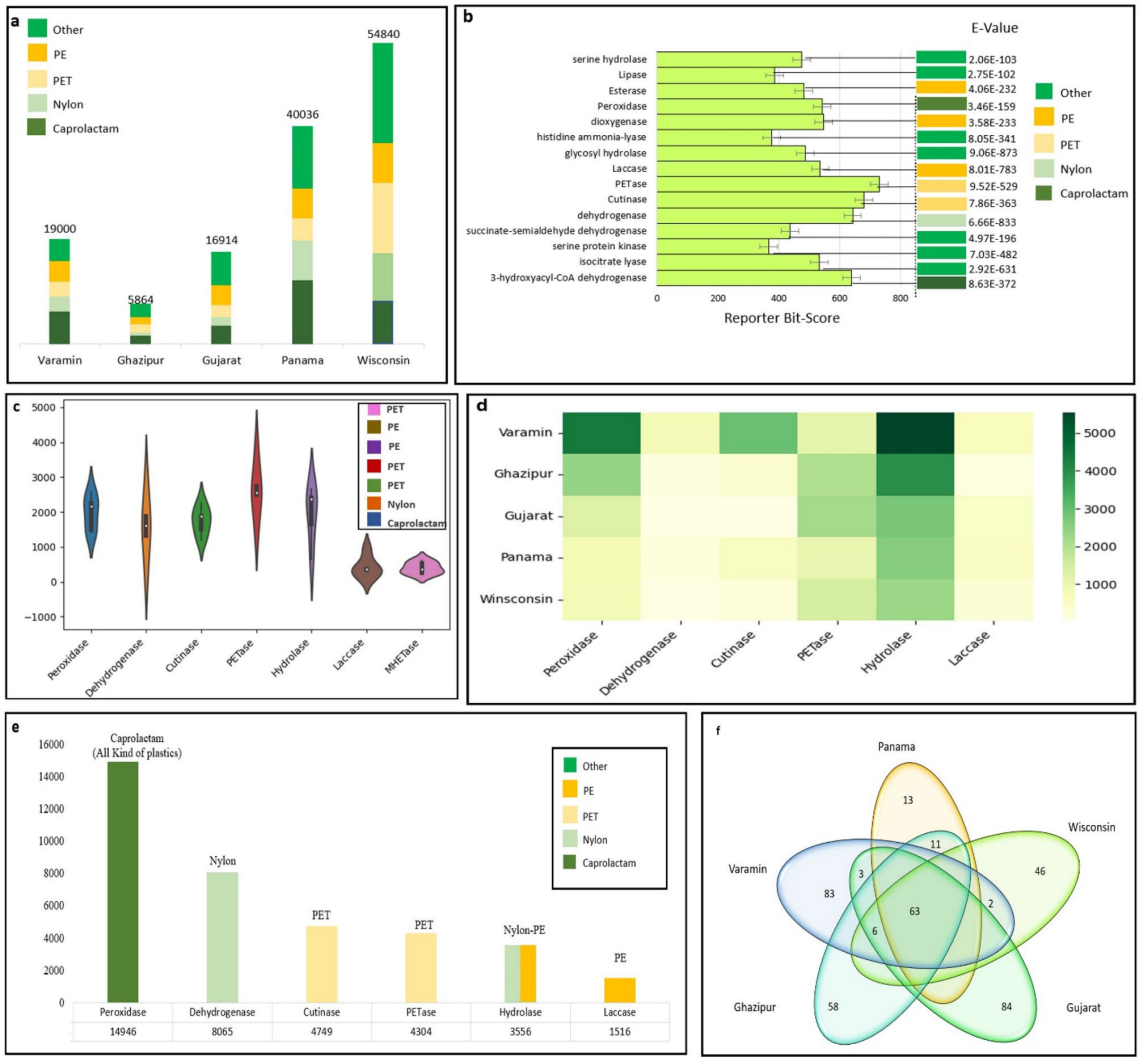
Profiling of predicted plastizymes in the PDEC. (**a**) Abundance of the predicted plastizymes in each sample. (Note that for each metagenome sample, the percentage of plastizymes was calculated for all samples.) (**b**) Reported bit score and e-value for putative plastizymes. (**c**) The violin plot shows the explainability of the samples in each province to interpret the first seven plastizymes. (**d)** Heatmap of the distribution of six plastizymes with high abundance in Varamin, Ghazipur, Gujarat, Panama, and Winsconsin samples separately. (**e**) Six plastizymes with the highest abundance in integrated PDEC. (**f**) Venn diagram analysis of five metagenome samples with different identified plastizymes groups.

The results indicate that the most abundant plastizymes belonged to Wisconsin samples, followed by Panama, Varamin, Gujarat, and Ghazipur samples. These plastizymes have been found to degrade caprolactam, nylon, PET, and PE. Other plastizymes include polyvinyl chloride (PVC), polyurethane (PUR), polyamide (PA), phenanthrene, and phthalates (Fig. 7a). The bit score and e-value of the selected putative plastizymes were also determined (Fig. 7b), with the highest bit score obtained for PETase (730), followed by cutinase (680) and dehydrogenase (620). These results suggest that PDEC are an excellent reference for PET and PE-degrading enzymes.

Focusing on the enzyme characteristics of the metagenomic sequences in plastic-contaminated environment, we first revealed the diverse distribution of plastizyme abundance (Fig. 7c) PETase had the highest abundance (average 2800) compared to other plastizymes, followed by hydrolases and peroxidases. In addition, we found that MHETase had the smallest redundancy (average of 500) among all five samples. Figure 7d illustrates the dispersion of the top six plastizymes in each sample. Hydrolase (nylon-PE), peroxidase, and cutinase enzymes were the most prominent plastizymes in the Varamin sample. In addition, hydrolase, peroxidase, and PETase were among the most abundant plastizymes in Gujarat and Ghazipur samples. In contrast to the Panama and Wisconsin samples, hydrolase was the main plastizyme with the highest quantity. Laccase and dehydrogenase were present in the lowest amounts in all samples. These results indicate that Varamin, Gujarat, and Ghazipur were more directly related to plastic particles than Wisconsin and Panama.

Also, Fig. 7e shows the six plastizymes with the highest abundance found in PDEC. The most abundant plastizymes were Proxidase, Dehydrogenase, Cutinase, PETase, Hydrolase, and Laccase. These plastizymes play important roles in Caprolactam, Nylon, PET, and PE.

Many plastizymes have rarely been described in metagenomic environments, because they have been identified in wet laboratories using traditional cultivation methods. In PDEC, we found all underrepresented plastizymes in the collected data. Strikingly, plastic-contaminated samples had 96 hydrolase plastizymes, 38 of which belonged to cutinase and PETase (PET-degrading enzymes), 15 to lipase (PE- and caprolactam-degrading), and the remaining enzymes belonged to nylon-, phenanthrene-, and phthalate-degrading enzymes. The second most crucial class of plastizymes is oxidoreductase. In PDEC, 27 different oxidoreductase plastizymes belong to laccases and dehydrogenases capable of PE, phenanthrene, and so on. In Fig. 7f the Venn diagram analysis revealed the presence of 63 plastizymes in all samples, as well as unique plastizymes in each sample, with the highest number of unique plastizymes found in Varamin (83), followed by Ghazipur (58), Gujarat (84), Panama (13), and Wisconsin (46). These results suggest that any plastic-contaminated site is a rich source of plastizymes and further research is needed to better understand their potential for plastic degradation.

**Figure 8:**
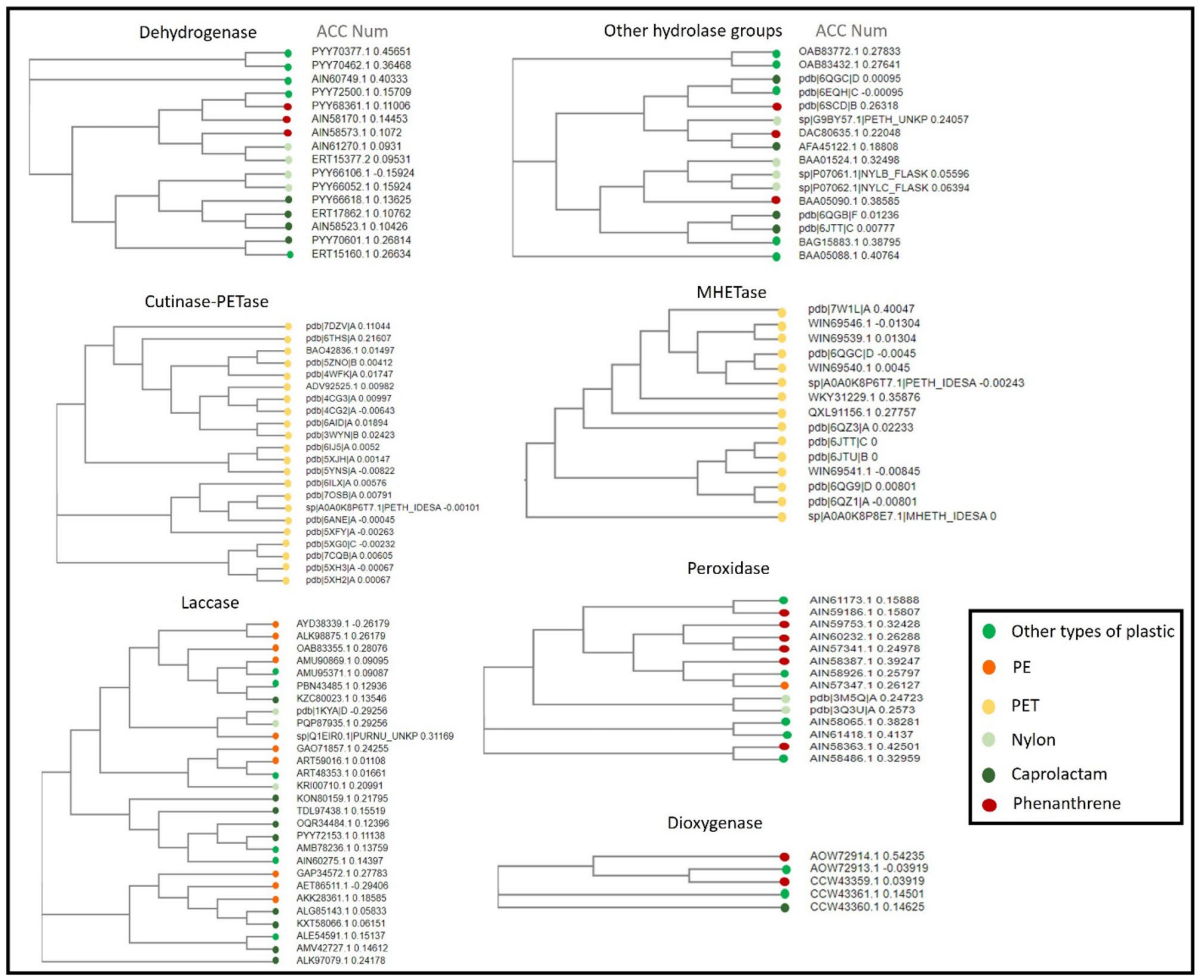
Seven phylogenetic trees of main Families of plastizymes that identified in PDEC. The hue of each node denotes the variety of plastic that the enzyme decomposes. They are downloaded from the NCBI RefSeq genomes.

The plastic-contaminated environments act as evolutionary repositories of plastic-degrading enzymes, as evidenced by the high diversity of plastizymes in the plastic-contaminated metagenome samples (Fig. 8). The depicted trees graphically elucidate the evolutionary connections among key plastizymes, encompassing the degradation of PE, caprolactam, nylon, PET, and phenanthrene, all within the framework of the comprehensive plastic-contaminated environment catalog (PDEC). In our analysis of metagenomic data, we observed many plastizymes previously identified experimentally. This could be attributed to the integrated different plastic-polluted environments exposure to a diverse array of plastics and chemicals. Further research on the underrepresented plastizymes in these samples could greatly expand our understanding of plastic contamination. Most of the enzymes identified were not previously identified by experimental approaches, suggesting that computational methods can be used to identify novel plastizymes. These findings have important implications for the field of metagenomics as they suggest that computational methods can be used to identify novel plastizymes and improve the accuracy of plastizyme identification.

## Conclusions

The current plastic recycling process is inefficient and has detrimental effects on marine and terrestrial environments, as well as living organisms. To facilitate future studies related to the discovery of new plastizymes that can play an important role in the purification of plastic pollution, we developed a workflow and the first integrated catalog of plastic-contaminated samples to facilitate the exploration of genes, proteins, taxa, and enzymes associated with plastic degradation. We further conducted an in-depth analysis of the reconstructed genomes to identify novel plastizymes. Our findings suggest that plastic-contaminated environmental microbial communities have the potential to degrade plastic components and that these environments are full of microbial groups and plastizyme sequences that can be used to break down plastic. Further research in this area could revolutionize the plastic degradation industry by identifying novel plastic-degrading bacteria and plastizymes.

### Statistical analysis

Statistical analyses and visualizations such as all heatmaps were conducted in Python 3.11.0 and PyCharm 2022.2.4 environment, utilizing the Matplotlib and Seaborn packages. Additionally, the diversity of taxa and taxonomic phylogenetic trees were visualized using the Krona chart-2.8.1^76^ (https://github.com/marbl/Krona/wiki) and Interactive Tree Of Life (iTOL) v5^77^ (https://itol.embl.de/), respectively. Further information can be obtained from the corresponding author upon request.

## Data Availability

The gene, taxa, and plastic degrading catalogs generated in the present study are available on https://drive.google.com/drive/u/1/folders/1diw2DSZeuhFSM04jBG66YXMubcqdS-Ul. The obtained nucleotide sequences of agricultural land of Varamin were submitted to the NCBI Sequence Read Archive (SRA) under the accession number SRR23085642.
